# Response to: Are endothelial cell proliferation and mesenchymal transition as distinguishing characteristics of 3-week Sugen5416/hypoxia mice model?

**DOI:** 10.1093/cvr/cvad075

**Published:** 2023-05-12

**Authors:** Julie Rodor, Shiau-Haln Chen, Andrew H Baker

**Affiliations:** Centre for Cardiovascular Science, Queen's Medical Research Institute, University of Edinburgh, 47 Little France Crescent, Edinburgh EH16 4TJ, UK; Centre for Cardiovascular Science, Queen's Medical Research Institute, University of Edinburgh, 47 Little France Crescent, Edinburgh EH16 4TJ, UK; Centre for Cardiovascular Science, Queen's Medical Research Institute, University of Edinburgh, 47 Little France Crescent, Edinburgh EH16 4TJ, UK

We would like to thank the reader for their interest in our paper and for their thoughts on endothelial cell (EC) proliferation and endothelial-to-mesenchymal transition (EndMT) in the Sugen5416/hypoxia (SuHx) model of pulmonary arterial hypertension (PAH).

Our study reported no increase in proliferating ECs in SuHx PAH mice,^1^ which the reader disagreed with. They reasoned this was because the differentially expressed genes between control and PAH were associated with the gene ontology (GO) terms regulation of angiogenesis (GO:0045765), regulation of vasculature development (GO:1901342), and EC proliferation (GO:0001935). We believe that GO term analysis alone is insufficient for making conclusions about cell proliferation. The GO terms highlighted by the reader also contain genes that regulate those processes in either a positive or negative direction, or both. Importantly, angiogenesis involves an intricate coordination and balance of EC proliferation, sprouting, and migration. It is overly simplistic to assume that because angiogenesis is induced, ECs must therefore be proliferating. Various studies have also provided evidence showing that opposing EC proliferative and migratory responses could present together in different pathologies and mechanistic contexts.^2–4^ The reader also suggested upregulation of *Bmpr2*, caveolin (*Cav*) 1/2, and secreted protein acidic and cysteine rich (*Sparc*) in PAH as markers of EC proliferation. This not only conflicts with our observation of *Bmpr2* downregulation in PAH,^1^ but also with the expression plot in their letter and findings in the wider PAH field of reduced BMPR2 signalling,^5^ EC hyperproliferation in *Cav1* and *Cav2* knockout mice,^6^ and unclear contributions of SPARC in proliferation and angiogenesis.^7^ Furthermore, we do not believe they are as direct or established a marker of proliferation as cell cycle–related genes (e.g. *Mki67*, *Pcna*, and *Mcm* genes). We are thus confident in our finding, which is based on robustly assessing established proliferation/cell cycle markers and the discrete cell cycle phases of each EC.

In their letter, the reader raised questions about our observation of limited EndMT in control and SuHx-induced PAH, reporting very high levels of Acta2 + control ECs in their analysis of murine whole lungs. We do not expect ECs to express any mesenchymal markers in control. As such, the 24% of Acta2 + control ECs they reported seem unexpectedly high, unlike the 1% Acta2 + control ECs we observed,^1^ though it is unclear from their plot label if it is 24% or 0.24%. Their analysis also showed control and intermittent hypoxia having similar proportions of Acta2+ ECs. This conflicts with their claim and matches our finding, which they had disputed. Although the reader provided immunofluorescent staining of PECAM1 and ACTA2 in lung cryosections from mice in normoxia or SuHx, there was lack of quantification of double-positive cells and statistical evaluation of the difference between groups. Furthermore, they have only evaluated one EndMT marker, ACTA2, where we know that a suite of markers should be used.^8^

EndMT is in itself a highly complex phenomenon and field of study, and Kovacic *et al.*^8^ have extensively reviewed the challenges posed by a lack of standard molecular definition. We used a panel of genes that could mark the following EndMT characteristics: reduced endothelial phenotype, increased mesenchymal phenotype, and increased expression of EndMT mediators.^1,8^ Our comprehensive assessment did not reveal any EC populations distinctly expressing these markers, suggesting EndMT was not present in our data. We also have no reason to believe that the tamoxifen induction efficiency and the fluorescence-activated cell sorting strategy we used are limitations in this context. EndMT has previously been studied in tamoxifen-induced condition.^9^ We found 95.5% of *TdTomato^high^* cells in our *Cdh5-CreERT2-TdTomato* mouse line, which were also *Cdh5^high^* and *Pecam1^high^* and were independently annotated as ECs by *SingleR.*^1^ Considering how EndMT could present as a transient phenomenon^9^ and how scRNA-seq only reveals a static snapshot of the cell state, we acknowledge that it is possible for EndMT to have occurred in earlier stages of our model, but this was not captured in our study. Importantly, we would like to emphasize that EndMT in PAH, and the wider cardiovascular disease field, has thus far been largely observational (i.e. being associated with a disease) and not causal.^8^

The final point raised by the reader was that our functional validation of CD74 should have focused on its immune function, rather than its impact on EC proliferation and barrier integrity. They also proposed using pulmonary artery ECs, instead of human umbilical vein ECs (HUVECs). PAH is a complex disease that involves not only an immune component, and the immune contribution of CD74 in ECs has been previously studied.^10^ However, the exact contribution of CD74 to the non-immune components of EC dysfunction in PAH remains unclear. We thus chose to extend our investigation of *CD74* beyond inflammation, through targeted assays of EC proliferation and barrier function, with HUVECs as an initial *in vitro* model system, before extending these findings further into other EC types and ultimately into *in vivo* models.

## Data Availability

The data are available through our published manuscript https://doi.org/10.1093/cvr/cvab296.
